# Paradox of Protective Behaviors Among Muslim Men During the Early Stage of the COVID-19 Pandemic in Aceh, Indonesia

**DOI:** 10.1017/dmp.2021.110

**Published:** 2021-04-06

**Authors:** Febri Nurrahmi, Tabsyir Masykar, Harapan Harapan, Tanzir Masykar

**Affiliations:** 1 Department of Communication, Faculty of Social and Political Sciences, Universitas Syiah Kuala, Banda Aceh, Aceh, Indonesia; 2 Department of Qur’anic Sciences and Tafseer, Sekolah Tinggi Agama Islam Negeri Tgk Chik Dirunding, Meulaboh, Indonesia; 3 Medical Research Unit, School of Medicine, Universitas Syiah Kuala, Banda Aceh, Aceh, Indonesia; 4 Tropical Disease Centre, School of Medicine, Universitas Syiah Kuala, Banda Aceh, Aceh, Indonesia; 5 Department of Microbiology, School of Medicine, Universitas Syiah Kuala, Banda Aceh, Aceh, Indonesia; 6 Akademi Komunitas Negeri Aceh Barat, Meulaboh, Indonesia

**Keywords:** COVID-19, mosque, Muslim, protective behaviors, risk

## Abstract

**Objective:**

The imposition of protective health protocols in public spaces to curb the spread of coronavirus disease (COVID-19) has confronted the ritual of congregational prayers in mosques for Muslims. This study examines the adoption of protective behaviors in the early stage of the COVID-19 outbreak and the influence of religion on risk perception by comparing precautionary behaviors in public and in mosques.

**Methods:**

Data were collected through an online survey of 327 Muslim men across the Aceh Province, Indonesia, from April 21, 2020, to May 2, 2020. The Wilcoxon signed-rank test and the paired t-test were employed to compare the uptake of protective behaviors in public and mosques.

**Results:**

The adoption of protective behaviors was higher in public rather than in mosques. It further revealed that the understanding of Islamic teachings during the pandemic has influenced perceived risk and the way Muslim men comply with the protective guidelines. Those who have complete, incomplete, or no compliance of precautionary behaviors have their own interpretation of Islamic teachings that inform their individual actions to manage the risk.

**Conclusion:**

This study suggests the significance of religious views for developing public health preparedness during the current and future pandemics in Aceh and other Muslim majority regions.

## Introduction

On March 2, 2020, the first 2 cases of the 2019 coronavirus disease (COVID-19) were reported in Indonesia, and, as of December 6, 2020, 575 796 confirmed cases and 17 740 deaths have been reported.^1^ This figure has positioned Indonesia as the country with the highest number of deaths from COVID-19 in Southeast Asia.^2^ The Indonesian Government has imposed strategic measures to curb the spread of the virus, and one of the first steps the government took was to encourage social distancing, followed by hygiene advice, such as wearing a face mask in public and frequent handwashing.

The rigorous imposition of social distancing will undoubtedly affect the Muslim daily congregation prayers and Friday prayers in the mosque. While only a small number of Muslim men perform daily congregational prayers, Friday prayer attracts large mass gatherings to mosques since the majority of Muslim men attend the prayer once a week. Praying in a congregation in the mosque is obligatory for every adult male Muslim,^3^ while they shall not consecutively miss Friday prayers more than 3 times for no religiously legitimate reasons.^4,5^

In support of the government’s instigation, the Indonesian Ulema Council (MUI), Indonesia’s top Muslim clerical body and a government-funded advisory body comprising a range of Indonesian Muslim organizations that produce *fatwa* (an advisory legal opinion by qualified Islamic jurists on Islamic law),^6^ issued a decree on how to practice daily communal prayers and Friday prayers during the COVID-19 pandemic. The decree states that those who live in the area with higher risk to the virus exposures may substitute Friday prayer with *Dhuhr* (early afternoon) prayers at home while those who are otherwise are allowed to perform Friday prayer in mosques.^7^ MUI has further encouraged people to avoid physical contact, bring their own prayer mats, and wash hands frequently.^7^ Following MUI, the Indonesian Ministry of Religion also issued the similar guidelines on protective measures in all places of worship. However, holding mass prayers with physical distancing and wearing face masks while praying would invalidate the prayers.^8^ Accordingly, in a normal condition, the congregation stand shoulder to shoulder in the row and do not cover their mouths.

Hence, the requirement to adjust to new religious norms is somewhat confusing and challenging for Muslims, especially in Aceh where Islam is a crucial part of their identity and culture.^9^ Aceh, known as the *Serambi Mekkah* (Veranda of Mecca), has been recognized as the most devoted region to Islamic faith and practice in Indonesia.^10^ More than 4 million or 98.19% of the total population are Muslims who profess strict adherence to orthodox, Sunni Islam.^11^ Aceh is the only province in Indonesia granted a right to practice Islamic jurisprudence.^12^

The first case of COVID-19 in Aceh was reported on March 26, 2020, and Aceh initially recorded the lowest number of COVID-19 cases in Indonesia. The way Aceh was dealing with COVID-19 was appreciated by the Indonesian Task Force for COVID-19 by asking other provinces to follow Aceh’s footsteps in curbing the spread of COVID-19. Later, however, the COVID-19 cases in Aceh began rising exponentially and it was ranked 15th out of 34 provinces based on COVID-19 case numbers.^1^ As of December 7, 2020, the number of reported cases was 8426, with 322 deaths.^13^

To combat the current COVID-19 pandemic, the involvement of the public is critical and everyone needs to fully participate by performing precautionary behaviors.^14^ Therefore, it is essential to find out the predictors of such behaviors. Previous studies highlighted the importance of perceived risk in motivating the engagement of protective behaviors during the pandemic.^15–20^ A study in Hong Kong showed that high levels of perceived susceptibility to COVID-19 were followed by high levels of protective behaviors.^15^ In Singapore, the low level of compliant behaviors with social distancing was associated with the low level of perceived risk.^16^ In a similar vein, a study in South Korea also showed that precautionary behaviors, including practicing hand hygiene, wearing a face mask, postponing or canceling social events, and avoiding crowded places, were significantly and positively associated with perceived risk and efficacy beliefs.^19^ A study in 10 countries across Europe, America, and Asia found that the adoption of protective behaviors during the pandemic, such as washing hands, wearing a face mask, and physical distancing, was correlated significantly with risk perception in all 10 countries.^20^ Nonetheless, perceived risk of COVID-19 in Indonesia was relatively low.^21^ Similarly, another study found that nearly 40% of the respondents from 7 provinces in Indonesia, including Aceh, had zero perceived risk to be infected with COVID-19.^22^ This would likely contribute to the low level of adherence to protective behaviors against COVID-19.

The objective of this study is to examine the adoption of protective behaviors in Aceh and to compare such behaviors in the public and mosques. It then also investigated motives of compliant and non-compliant behaviors to understand the significance of religion on behavioral changes within Muslim communities during the pandemic in Aceh, Indonesia. This study adopted the Health Belief Model to explain the uptake of protective behaviors. Among other health models, this model is more appropriate to this study since it has initially been used to explain preventive health measures such as uptake of screening tests or immunization.^23^ This model proposes that perceived risk, together with perceived benefits and barriers, is a significant predictor of behavioral changes.^24^ This study further expanded the Health Belief Model by taking into account religion. Previous studies have tested that religion has reduced^25^ or increased fear of death,^26^ and inhibited fear of crime.^27^ To date, previous studies on the adoption of protective behaviors in Aceh^28,29^ did not raise the issue of an individual’s Islamic understanding toward the pandemic. In fact, it is pertinent to discuss religion in the context of Aceh, considering Islam as a core element in Acehnese society.

## Methods

### Study Setting

Data collection was conducted through an online survey using Google Forms between April 21, 2020, and May 2, 2020, 2 months after the first confirmed COVID-19 case was reported. The survey was distributed on WhatsApp, the most used communication platform in Indonesia, with about 40% of Indonesians using WhatsApp.^30^

The participation was voluntary. Interested people were asked 4 screening questions to assess their eligibility before undertaking the survey. They were required to confirm whether they were Muslim, male, living in Aceh, and over 15 years of age. Those considered ineligible by these questions were then terminated from participating in the survey. This study targeted Muslim men since they are obliged to perform congregational prayers and Friday prayers in mosques. Furthermore, 15 years old is the end of the age of puberty for Muslim boys to be considered as *baligh* (mature) and then required to perform the 5 daily prayers and Friday prayers.^31^

Those who passed the online screener were given the overview of this study, followed by a mandatory consent question. Only respondents who gave their informed consent were able to proceed to the survey. The estimated total population for the study was approximately 1.6 million people.^32^ Thus, the minimum sample size was 385 based on a 5% margin of error and a confidence interval of 95%.

### Study Instrument

The survey used a semi-structured questionnaire that included closed-ended and semi-closed questions. Semi-closed questions provided the respondents with not only answer options, but also enough space to express their own opinions.^33^ A questionnaire was developed, tested among a small group of pre-testers, and was revised prior to the actual study. The questionnaire comprised 4 parts. The first part reviewed the respondents’ demographics, including district, age, educational attainment, and employment. This study was anonymous since it did not ask participants to provide their names in the survey. The second part addressed questions to congregational prayers in mosques. This part also included 4 semi-closed questions regarding the reasons and feelings for attending and not attending congregational prayers in mosques. The semi-closed questions allowed respondents to provide their own responses if none of the given answers suited them. The third part consisted of questions on protective behaviors against COVID-19 in mosques. The last part was questions on general protective behaviors against COVID-19. The third and fourth parts were developed and modified from primary prevention introduced by the Indonesian Health Ministry, such as physical distancing, wearing a face mask in public, and washing hands after going outside. Data are stored in the Universitas Syiah Kuala research drives and a password is required; thus, only the principal investigator has access to the data.

### Statistical Analysis

The Wilcoxon signed-rank test or the paired t-test was used to assess whether the 2 sets of scores that came from the same respondents (paired data) are statistically different.^34^ The Wilcoxon signed-rank test was commonly used for ordinal-scale data,^35^ whereas the paired t-test was appropriate to analyze interval-scale data.^36^ The Wilcoxon signed-rank test was used to compare the mean rank between the crowd avoidance and attendance of congregational prayers. The paired t-test was employed to compare the mean differences between the frequency of daily congregational prayers before and during the pandemic, wearing a face mask in public and in mosques, and the handwashing after returning from outside and mosques. The Kruskal–Wallis test was also conducted as an additional statistical analysis to examine attendance of mass prayers in mosques by age, education, and employment status. All statistical analyses were conducted using SPSS software, version 26 (IBM Corp, Armonk, NY).

## Results

### Characteristics of the Respondents and Their Protective Behaviors in Public

This study received 380 responses; 53 of them were excluded due to incomplete data, and 327 questionnaires were proceeded for further analysis. This sample size was deemed sufficient to represent the population of the study since it had a 5.42% margin of error that typically ranges from 3% to 10% at a 95% confidence interval.^37^ Out of the 327 respondents, nearly half were from Aceh Barat (43.1%), followed by Banda Aceh (19.6%), and Aceh Besar (11.6%) (Table 1). The distribution of the respondents based on districts covered 18 out of 23 cities and regencies in Aceh Province.^38^ In addition, almost half of the respondents were of ages 31–40 (45.6%), followed by 21–30 (30%), 41–50 (13.8%), 15–20 (6.4%), 51–60 (3.7%), and above 60 years (0.6%). Approximately 41% of respondents held undergraduate degrees, 38.8% had postgraduate degrees, and 16.5% graduated from senior high schools. More than half of the respondents were government employees (52.6%), followed by self-employed (15.3%), students (14.7%), and private employees (11.3%).

**Table 1.** The respondents’ characteristics (n = 327)

Variable	Frequency	Percentage
District		
Aceh Barat	141	43.1
Aceh Barat Daya	7	2.1
Aceh Besar	38	11.6
Aceh Jaya	4	1.2
Aceh Selatan	6	1.8
Aceh Singkil	2	0.6
Aceh Tengah	4	1.2
Aceh Timur	1	0.3
Aceh Utara	6	1.8
Banda Aceh	64	19.6
Bener Meriah	1	0.3
Bireuen	4	1.2
Langsa	3	0.9
Lhokseumawe	6	1.8
Nagan Raya	18	5.5
Pidie	4	1.2
Sabang	2	0.6
Simeulue	6	1.8
No answer	10	3
Age (year)		
15–20	21	6.4
21–30	98	30
31–40	149	45.6
41–50	45	13.8
51–60	12	3.7
> 60	2	0.6
Education		
Primary school	2	0.6
Junior high school	2	0.6
Senior high school	54	16.5
Diploma	5	1.5
Undergraduate	137	41.9
Postgraduate	127	38.8
Employment		
Government employee	172	52.6
Private employee	37	11.3
Self-employed	50	15.3
Student	48	14.7
Other	20	6.1

**Figure 1.**
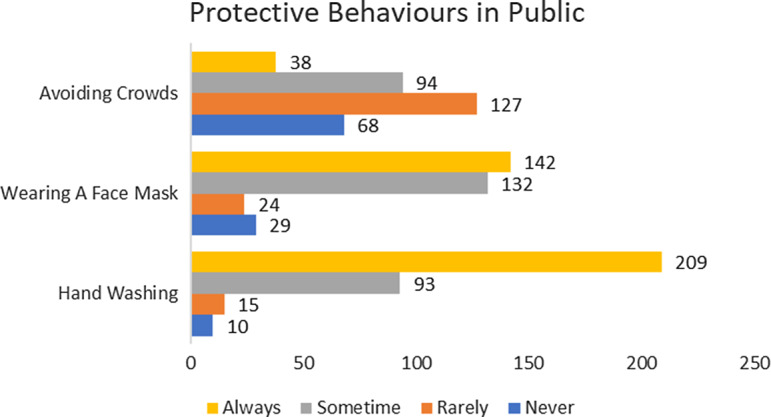
Protective behaviors in public.

Among 3 preventive measures, wearing face masks and washing hands were frequently adopted. The proportion of respondents who preferred to always or sometimes use a face mask accounted for 84%, whereas those who rarely or never used a face mask only made up 14% of the total sample (Figure 1). Moreover, more than half of the respondents (64%) always washed their hands after visiting public places, followed by 28% who sometimes took this measure. The adoption of social-distancing measures was moderate. Respondents said that they rarely (39%) and sometimes (29%) avoided congregating in public. About 21% of respondents showed non-compliant behaviors to the Indonesian Government’s recommendation to avoid crowds during the COVID-19 outbreak. Only 11% of the respondents demonstrated complete compliant behaviors by always avoiding crowds (see Figure 1).

### Comparison of Protective Behaviors in Public and Mosques

Most respondents (68%) said that they rarely or sometimes avoided public places. In a similar vein, respondents also showed a tendency to disregard such a behavior in relation to congregational prayers. Most respondents still attended Friday prayers (84%) and daily congregational prayers (79%) in mosques. Among those who performed communal prayers, 39% of the respondents always performed mass prayers in mosques, whereas 30% said they prayed only 1–2 times a day in mosques. The Wilcoxon signed-rank test revealed that there was a statistically significant difference between the frequency of crowd avoidance and the attendance of prayers in mosques (*z* = −10.072, *P* < 0.001). The result further suggested that respondents were more likely to avoid crowds than communal prayers in mosques. A further analysis using the Kruskal–Wallis test showed that there was no significant difference between the attendance of congregational prayers in mosques by age, education, and employment status (*P* > 0.05) (Supplementary 1).

The majority of respondents said that they always (44.4%) or sometimes (36.1%) used face masks when going to public places (Table 2). The remaining respondents confirmed that they never (11.3%) or rarely (8.2%) wore face masks in public. In contrast, most of the respondents said that they never wore a face mask in mosques (55.3%), whereas those who always used a face mask in mosques were at the lowest (8.6%). There was a statistically significant difference between the frequency of wearing a face mask in public (mean: 2.05 ± 0.95) and in mosques (mean: 0.86 ± 01.06): *P* < 0.001 (see Table 2). These results suggested that the use of a face mask was preferred in public, in general, rather than in mosques.

**Table 2.** Comparison of wearing a face mask and handwashing in public and in a mosque

Protective Behavior	Frequency Category (%)				**Mean** **(± SD)**	t	***P*** **-value**
	Never	Rarely	Sometimes	Always			
Wearing a face mask						-18.21	<0.001*
In public	29 (11.3)	21 (8.2)	114 (44.4)	93 (36.1)	2.05 (0.95)		
In a mosque	142 (55.3)	32 (12.4)	61 (23.7)	22 (8.6)	0.86 (1.06)		
Handwashing						-4.94	<0.001*
In public	10 (3.9)	14 (5.4)	87 (33.9)	146 (56.8)	2.44 (0.77)		
In a mosque	19 (7.4)	23 (9)	98 (38.1)	117 (45.5)	2.21 (0.89)		

*Notes:*

* Analyzed using paired t-test; SD = standard deviation.

Most respondents (56.8%) said they always washed their hands after returning from public spaces and nearly half (45.5%) took the same measure after going to mosques (see Table 2). A comparable proportion of respondents, 38.1% for mosques and 33.9% for public spaces, opted for “sometimes” for this safety measure, whereas the difference between the mosques and public place group for “always’’ category was small, with the difference of only 29 respondents between the groups. There was a statistically significant difference between the frequency of washing hands after being in public (mean: 2.44 ± 0.77) and in mosques (mean: 2.21 ± 0.89): *P* < 0.001. The results implied a greater tendency to wash hands after returning home from public places rather than after praying in mosques.

### Reasons for the Adoption and Rejection of Protective Behaviors

**Figure 2.**
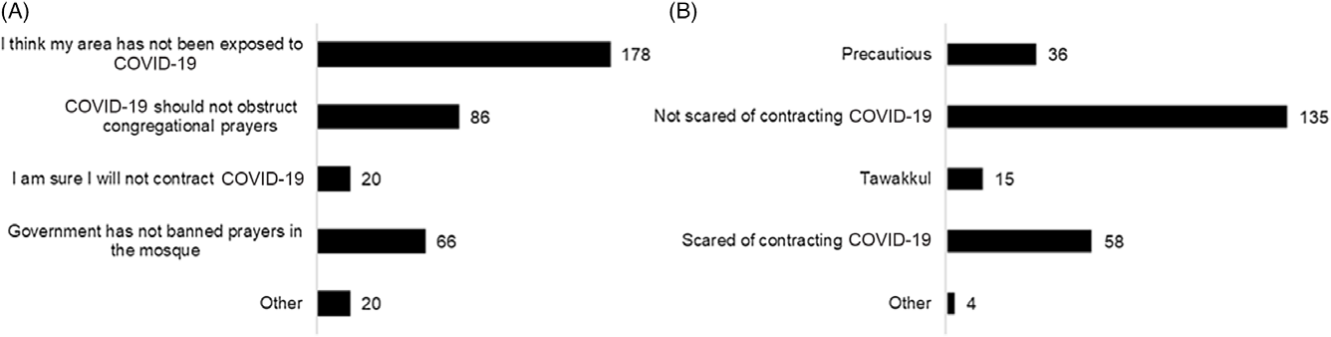
Reasons for attending congregational prayers (A) and feeling during congregational prayers in mosques (B).

Most respondents expressed that they still went to mosques to pray since they believed that their area was free from COVID-19 (Figure 2A). Moreover, some respondents agreed that COVID-19 should not obstruct them from prayers. Other respondents still performed congregational prayers because the district administration did not ban prayer in mosques. Finally, only 20 respondents were confident enough to answer that they would not contract COVID-19.

The analysis of the open-ended answers could possibly offer further explanation. First, they considered that congregational prayers in mosques are obligatory regardless of the circumstances. One respondent stated, “I fear the creator of [the] virus more than the virus.” Another said, “There is no good or bad will befall a person but permission from Allah.” Second, a mosque was considered the holiest place since Muslims perform *wudu* (a ritual washing of their bodies to get rid of the minor impurities) before praying. Third, 2 people admitted that congregational prayers were part of their daily habits. They did not feel comfortable if they did not go to mosques to pray since they were used to it.

In addition, 4 people expressed that they did prayer with high precaution. They performed protective behaviors, including physical distancing when praying, wearing a face mask, and washing hands.

In response to a question on feeling during congregational prayers in mosques, 54% of the respondents said that they were not afraid of contracting COVID-19, whereas 23% of them stated otherwise (see Figure 2B). Apart from those 2 given options, 2 themes emerged from open-ended answers, including precaution (15%) and *tawakkul* (trust and reliance on Allah) (6%). Those who mentioned *tawakkul* believed that everything that happens in their life is determined by Allah, so they would accept whatever may turn out to be. One respondent stated, “I am sure Allah always keeps his servants who worship him.” In this sense, their religiosity has managed their fear of COVID-19. Another said, “I am afraid of harm, so I am trying to get to the mosque to pray in congregation to bring good.”

**Figure 3.**
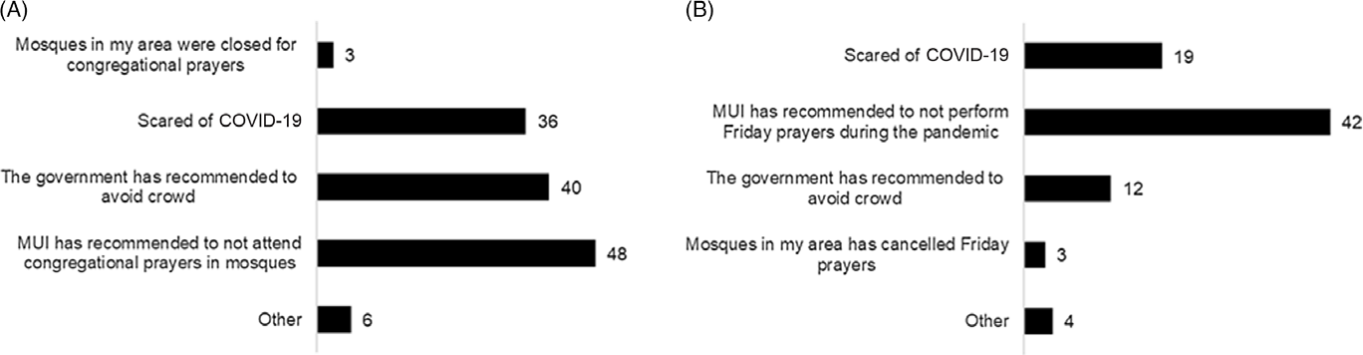
Reasons for not attending daily communal prayers (A) and Friday prayers (B) in mosques.

For a question on the reason for no longer attending prayers in congregation, the most common answer was that the MUI had recommended doing so (Figure 3A). In this context, respondents showed obedience of the *ulama’s fatwa*, followed by obedience of the government’s measures to avoid crowds, and fear of contracting COVID-19. Similar responses were found for a question of why they no longer performed Friday prayers (see Figure 3B). Furthermore, the option “other” revealed that few respondents gave up their prayers in mosques as they followed the *Sunna* (teachings of Prophet Muhammad). One respondent cited the word of Prophet Muhammad, “Flee from the leper (contagious disease) as you would from the lion.” In this sense, they considered COVID-19 a contagious disease from which they should be running away.

## Discussion

### Paradox of Protective Behaviors

Our study indicated that the uptake of protective behaviors was relatively moderate to high among Muslim men of age 15 years in Aceh. However, the adoption of social distancing by that group was much lower when compared to other preventive measures. This could be explained by that social distancing measures contradict a religious ritual within Acehnese society to reject the plague. During the COVID-19 outbreak, many Acehnese gathered in places of worship or open fields to hold mass prayers to avoid harm.^39^ The second possible explanation of why the adoption of social distancing was much lower is that social distancing during COVID-19 could lead to anxiety/depression and household income loss.^40^ This finding is contrary to previous studies, which suggested that social distancing was frequently adopted in Aceh,^28^ Indonesia,^41^ and other collectivist societies, such as China,^42^ during the early stage of the pandemic.

Moreover, the findings suggested the paradox of protective behaviors. While Muslim men over age 15 years in Aceh were more likely to practice safety measures when they are in public spaces, they eased their measures when they are in mosques. The results of this study further suggested 3 different groups of Muslim men in Aceh in seeing congregational prayers and preventive measures in mosques. There are those who showed a non-compliant behavior (risk-taking behavior) by praying in mosques, those who showed incomplete compliance (moderate risk-taking behavior) as a result of negotiation between their praying rituals and protective behaviors through the precautionary behaviors in mosques, and those who showed a complete compliant behavior (risk-avoidance behavior) by canceling their congregational prayers in mosques, either daily or Friday prayers.

#### Risk-Taking Behavior

Those who attended prayers in mosques took risky behaviors. The analysis of the reasons for performing such risky behavior revealed that those who performed daily prayers perceived the risk as low. This may be explained by the low numbers in confirmed COVID-19 cases in Aceh. Aceh was not the COVID-19 red zone; only 13 confirmed cases of COVID-19 were found by the time of the study.^13^ This is in line with the majority of respondents who reported that they were not afraid of contracting COVID-19. However, findings also demonstrated that those who performed congregational prayers were more likely to avoid crowds. In this sense, the second most common answers may explain such a paradox. Few respondents agreed that COVID-19 should not obstruct them from prayers. Their faith to follow Allah’s rule has put aside the potential risk of contracting the virus in mosques. In addition, the open-ended answers revealed that congregational prayers were performed to avoid harm and bring good during the pandemic. In this regard, using the Health Belief Model, there are 3 possible explanations. First, respondents’ religiosity has lessened their perceived risk or fear of getting infected by COVID-19. Second, their understanding of Islamic teachings has become their perceived barrier to avoid praying in mosques. Third, they believed mass prayers in mosques brought perceived benefit to avoid harm from COVID-19.

Furthermore, a highlighted finding was that they still performed prayers in mosques as it is part of their daily ritual. This showed that devout people were not easily persuaded to adapt their religious rituals in response to the COVID-19 outbreak. This is in line with Istratii^43^ who argued that most non-Western communities that exercise religious tradition as part of their life and identity do not agree with the idea that religions can be easily adapted during special circumstances, such as the current pandemic. This appears to be the case in Aceh. Apart from the official implementation of Sharia in the region, Aceh has practiced Islamic culture.^10^

Apart from the low-perceived risk, the results also demonstrated that many respondents insisted on praying in mosques because district administrations did not ban prayers in mosques. This also reflects the contradiction between regulations issued by the Indonesian Government and the provincial or district administration. While the Ministry of Religious Affairs and MUI have advised Muslims to worship from home, the district administration and the Aceh Ulema Council (MPU) allowed people to perform daily mass prayers. Unclear and inconsistent government risk measures during the pandemic may confuse members of society and allow them to interpret the situation on their own.^44^

#### Moderate Risk-Taking Behavior

Few respondents expressed that they did prayers with high precautions. Such a finding corresponds to that some respondents were precautious during congregational prayers. This also indicated that even though they did not abide by the government’s measure to not visit public places such as mosques, they had enough confidence to prevent themselves from contracting the virus by performing protective behaviors, including physical distancing when praying, wearing a face mask, and washing hands.

However, the paradox of protective behaviors was also evident in wearing a face mask. While reporting high frequency in wearing a face mask in public, many respondents were not inclined to do so in mosques. It may be explained by the fact that wearing a face mask is forbidden during prayers.^8,45^ While they were able to use a face mask on their way to and from mosques and took it off during a prayer, face mask use may be considered useless since the mask should be taken off when the prayer starts. Moreover, a previous study on face mask use in 2 countries, China and Poland, indicated that using face masks were affected by cultural views and practices.^46^ As wearing face masks was not a common practice in mosques during prayers, it was more unlikely for Muslim men in Aceh to wear face masks in mosques. In this sense, people were more likely to perceive a strong barrier to wearing a face mask. However, there was a view that wearing a face mask during the pandemic is allowed.^8,45^ Therefore, only those who believed that face masks were allowed to be used during prayers and that it could help to protect them from contracting the virus were more likely to wear a face mask in mosques.

Unlike wearing a face mask, hand washing was more likely to be performed. It is assumed that Muslims get used to cleaning their bodies 5 times a day. Thus, it is not difficult to develop a new habit of washing hands with soap. In this context, the perceived barrier was low. They did not need to spend more energy to adapt to this new habit as it resembles an older habit.

#### Risk-Avoidance Behavior

The analysis of those respondents who canceled their daily mass prayers or Friday prayer showed their obedience to the MUI to not perform daily congregational prayer and Friday prayer. This finding resonates with Mushodiq and Imron^47^ in that MUI plays a significant role in mitigating the spread of COVID-19 by promoting a new model of worship for Muslims during the outbreak. Moreover, perceived risk was another reason. Consistent with the previous literature on perceived risk as the key predictor in social distancing,^16–20^ the current study found that those who had a higher perceived risk to be infected with COVID-19 were more likely to avoid communal prayers in mosques. This study also documented that some respondents had religious knowledge about what Islam has taught to be done during a pandemic. Such knowledge has further inspired them to stop their communal prayers in mosques for the greater good.

This study's findings also complement the existing evidence in Aceh^28,29^ by taking into account the religious aspect to explain the behavioral responses during the pandemic. Considering the piety embedded in Acehnese society, this study’s findings may provide an explanation for the adoption of protective behaviors, in general, not only constrained to worship activities. Thus, this study may yield more insights about the adoption of protective behaviors in Aceh and other Muslim communities.

This study has shown that adaptation to new religious norms is challenging for Muslim men. Therefore, the government should strengthen the role of the *ulama* and Muslim clerical councils in preaching to Muslims to obey *ulama’s fatwa,* preventive health measures, and government instruction. In addition, as suggested by an earlier study, such preaching would be more effective to be delivered through online newspapers and social media.^48^ This is important to curb the spread of COVID-19 and protect the whole community.

### Limitations

There are some limitations of the study. The main drawback of this study relies on the way the questionnaire was distributed. As this study used the WhatsApp platform, the respondents participated through invitation via WhatsApp contacts. Therefore, it was possible that this survey did not reach a large number of people. To minimize such selection bias, 6 persons living in different parts of Aceh were employed to distribute the survey link. It was expected to reach as many eligible people to participate in the survey. However, this online survey may miss potential people who were illiterate, were not familiar with WhatsApp, or did not have access to the Internet. Regardless of this limitation, such an online survey was the most feasible data collection method during the current pandemic. This study also did not collect the information on the number of people invited to participate in the survey. Therefore, the response rate of this online survey cannot be provided. Moreover, although our samples came from several districts in Aceh, they were not balanced on quota. Finally, the study focused on only perceived susceptibility of contracting COVID-19 and perceived barriers that prevent someone from adopting protective behaviors. This study did not assess perceived severity and perceived benefit that are mentioned in the Health Belief Model. However, the concept of perceived benefit as one of the factors influencing such risk-taking behaviors was also discovered from open-ended answers.

## Conclusions

This study has shown how the different interpretations of Islamic teaching during the pandemic result in risk-taking and risk-avoidance action. Based on the results of the study, different interpretations of Islamic teachings inform Muslim men to comply or not. First, those who still practice congregational prayers in mosques believe that the crisis shall not restrain their worship ritual since by doing so Allah will protect them. In this sense, religion has loosened their perceived risk of contracting the virus and acts as a religious barrier that prevents them from avoiding communal prayers in mosques. This explains why people still attend congregational prayers in mosques and ignore the possibility of contracting the virus. Second, there is a group of people who believe that they should pray in mosques while engaging with protective behaviors. These people may think that performing precautionary measures, such as wearing a face mask and washing hands, will lower their likelihood to contract the virus during congregational prayers in mosques. Third, some are certain that Muslims are required to make every effort to avoid the contagious disease during the outbreak. They have a high perceived risk on congregational prayers in mosques and they are not supposed to pray in mosques as it will cause more harm than good.

## Supplementary material

For supplementary material accompanying this paper visit http://dx.doi.org/10.1017/dmp.2021.110. click here to view supplementary material
