# Supplementary material for: Paradox of Protective Behaviors Among Muslim Men During the Early Stage of the COVID-19 Pandemic in Aceh, Indonesia
Source: Disaster Med Public Health Prep. 2021 Apr 6:1–8. doi: 10.1017/dmp.2021.110 (PMC8144824; doi:10.1017/dmp.2021.110)
Supplement: Supplementary file 1 [file S1935789321001105sup001.docx]

Supplementary 1. Comparison of the attendance of communal prayers in mosques

| Protective behavior | Kruskal Wallis  H | df | p-value |
| --- | --- | --- | --- |
|  |  |  |  |
| Age | 9.574 | 5 | 0.088 |
| Education | 8.802 | 5 | 0.117 |
| Employment | 5.241 | 4 | 0.263 |

* Analysed using the Kruskal-Wallis Test
